# Supplementary material for: An investigation into waste handler’s knowledge of management of isolation waste: A case study of Dr George Mukhari Academic Hospital, Gauteng, South Africa
Source: PLoS One. 2024 Jun 10;19(6):e0305224. doi: 10.1371/journal.pone.0305224 (PMC11164387; doi:10.1371/journal.pone.0305224)
Supplement: Attachment — Submitted filename: Plosone_revision.docx [file pone.0305224.s001.docx]

Revision: PONE-D-23-28839

Review A

| The paper presented information regarding the respondents' (represented by 34 respondents) awareness of waste handling and waste identification at Dr. George Mukhari Academic Hospital (DGMAH), Gauteng, South Africa. The authors claimed that there is a correlation between waste handling and the age (line 170) of the respondents and recommended specific training for a certain age bracket. Though waste handling is mostly on the hand of the waste management office of the hospital to include the janitors, crews, and lab technicians, but it was not presented in the methodology the current role of the respondents in the hospital. In line 97, the authors mentioned the demographic profile of the respondents but still, it lacks information about the respondents' current role at DGMAH. For instance, of course, if the respondent is a clerk then most probably the clerk does not have full knowledge as to how the waste in the hospital must be handled because it is not aligned with her/his work in the hospital. The manuscript though used the term waste handler which connotes a meaning that all 34 respondents are working as waste handlers at DGMAH | All waste handlers are involved in the collection of isolation waste. This has been included in the methodology as part of the purposive sampling (Page 4). |
| --- | --- |
| All tables should be revised.  Line 73. Should be DGMAH  Line 90 to 91. “Participants were recruited from November 11, 2019, until August 10, 2020…. “ When did the actual study start? Is it after August 10, 2020 (which is after the “recruitment” of participants? Does this further mean that the period starting November 11, 2019, to August 10, 2020, is dedicated to recruiting the participants?  Line 91. What is the importance of asking the participants to explain their daily duties to the researcher as a basis for the selection process? Lines 117 to 118. I do not understand this sentence.  Line 134 to 135. What particular work is referred to in these lines?  Line 159. In Table 2, what is meant by the column “all correct response, one correct response, and incorrect response”. Maybe the table can be restructured in a more informative way.  There are some entries such as “Isolation waste must be labelled or marked isolation” that is confusing.  ```Line 162 to 163. Do the authors mean, that the respondents’ age, gender, education, or experience are not statistically related to their knowledge or ability of waste handling?  Line 165. “Knowledge of waste handlers regarding the types of waste generated in the isolation unit was not significantly influenced by these demographic and professional factors…”. Do the results of the survey, from 34 respondents, show that knowledge of handlers on waste handling is not statistically influenced by their age, gender, education, or experience however these finding seems not acceptable and is a contradiction of reality. I have a doubt that the number of respondents (34) is not enough to arrive at this general remark.  Also, the scale of the response (0, 1, and 2) is very limited which may have affected the proper response of the respondents.  Line 172 to 173. The results show that the handlers’ age is not correlated with color identification. This is opposite to the result presented in line 170, where the waste handlers' knowledge of waste isolation is associated with age.  Line 183. Kindly revise Table 3 and present it in a simple yet informative one.  Line 191 to 192. “….only one correct response and incorrect responses…” Not clear.  Line 197. May be “importance” rather than “necessity”.  Line 203. Same comments for Table 4 as with Table 2.  Line 243. The discussion percey pf the manuscript is only a repetition of the information given in the Results. It is suggested to merge these 2 sections. | This have all been corrected.  Participants were recruited from November 11, 2019, until 4 December 2019.  Participants were asked to explain their daily duties to the researcher before taking part in the study the process was important to identify individuals who not only meet the criteria for the study.  This is the participants years of experience as a waste handler.  In Table 2, the coding of the response was structured in a way that the respondents  Isolation waste must be labelled or marked with the term isolation waste.  Yes.  The entire waste handlers in the hospital were 34. The survey included all the waste handlers. As such, the 34 respondents are representative of the entire waste handlers involved in the collection of isolation waste.  The result is not contradictory. While there was no association between age and knowledge of sharp objectives, and colour identification, there was, however, statistically significant difference with respect to their knowledge of isolation waste.  There is no other way to present Table 3 as it relates to association between the demographic and specific questions addressing their knowledge.  However, there were 7 respondents (20.6%) who provided only one correct response despite being allowed to select multiple answers. This tendency may indicate a potential limitation in the depth of their knowledge. Furthermore, 2 participants (5.9%) provided incorrect responses to the question.  This has been corrected.  The responses were categorized as correct response- when they waste handlers answers all question questions, incorrect- when they failed to answer any of the questions correctly, and one correct response- when they answered one correct response.  The comment is noted. The discussion sections discuss the results in relation to relevant literatures. The authors believe keeping both separate will be beneficial to the readers. |
