# Supplementary material for: An investigation into waste handler’s knowledge of management of isolation waste: A case study of Dr George Mukhari Academic Hospital, Gauteng, South Africa
Source: PLoS One. 2024 Jun 10;19(6):e0305224. doi: 10.1371/journal.pone.0305224 (PMC11164387; doi:10.1371/journal.pone.0305224)
Supplement: Attachment — Submitted filename: Plosone_revision.docx [file pone.0305224.s002.docx]

Revision: PONE-D-23-28839

Review 2

| 1. What were the specific findings regarding waste handlers' knowledge of waste types, color codes, safety precautions, and internal policies related to isolation waste management at Dr. George Mukhari Academic Hospital? | We do not understand in clear terms the reviewer comments. However, the findings are included in the abstract  The study found that the majority of waste handlers demonstrated a good understanding of waste types generated in the isolation unit, including sharps waste, human tissue waste, infectious waste, and general waste. They also correctly identified examples of sharp waste, such as injections, blades, glass slides, and needles. Additionally, most respondents were aware of the colour code used for representing infectious waste as "yellow" and "red." The study revealed a statistically significant association between waste handlers' age and their knowledge of isolation waste, suggesting that age may influence their understanding of waste management practices. Furthermore, experience was found to be significantly associated with waste handlers' knowledge of the health-hazardous nature of isolation waste. While the majority of waste handlers recognized the importance of wearing protective clothing and correctly marking isolation waste, some respondents were not aware of the internal policy for waste handling such as guidelines and protocols specific to the segregation, packaging, labeling, and disposal of waste generated within the isolation units (Page 1). |
| --- | --- |
| What are the potential health and environmental risks associated with improper management of medical waste, specifically isolation waste? | This has been provided:  Improper disposal of isolation waste presents significant risks to both healthcare workers and the environment. For instance, inadequate disposal of sharps waste, including needles and syringes, can lead to needlestick injuries among healthcare workers. Such injuries pose a serious risk of exposure to bloodborne pathogens such as HIV, hepatitis B, and hepatitis C (5). Similarly, improper disposal of isolation waste, such as dumping or inadequate landfilling, can have detrimental effects on the environment. Contaminating soil and groundwater with hazardous chemicals and pathogens from isolation waste poses risks to ecosystems and human health (6). (Page 2). |
| What are the critical steps involved in the management of isolation waste? | These are explained in page 2 line 57. For reference: The management of isolation waste involves several critical steps, including waste segregation, proper containment, safe transportation, and appropriate disposal (7). |
| Are there specific guidelines and regulations in South Africa for healthcare waste management? Are these guidelines consistently followed by waste handlers in different healthcare settings? | Yes, there are specific guidelines and regulations of medical waste in South Africa (The management of Healthcare Risk Waste (HCRW) is regulated by various legislation, including the National Health Act no. 61 of 2003, the National Environmental Management Act no. 107 of 1998, the National Environmental Management Waste Act no. 59 of 2008, and National Core Standards (10). These regulations establish comprehensive protocols governing the handling, storage, collection, transportation, treatment, and disposal of medical waste (Page 3).  However, these guidelines are not consistently followed by waste handles (Page 3). |
| What unique challenges related to waste management may Dr George Mukhari Academic Hospital face, and how do these challenges impact waste handlers' practices and compliance with isolation waste management protocols? | Given that compliance remains a challenge in many hospitals across South Africa, we assume there may be variations in knowledge, awareness, and adherence to waste handling guidelines among waste handlers in different healthcare settings including Dr George Mukhari is no exception. Hence, the need to investigate the level of knowledge and awareness of isolation waste among handlers in Dr George Muhkari Academic Hospital. This was informed as the hospital serves as a significant tertiary medical institution in the region. As a facility providing healthcare services to a diverse patient population, including those with infectious diseases. |
| Can you provide more information about the selection process for participants in the study? How were waste handlers purposively selected? | This is provided by including the following: The hospital management played a crucial role in participant recruitment by disseminating information about the study to the heads of departments and waste handlers in their respective departments. Participants were purposively selected from waste handlers stationed in hospital wards, specifically those responsible for handling isolation waste, including its removal, packaging, and sealing (Page 4). |
| How was permission obtained from the hospital management to contact the participants? Did the management also assist in the recruitment process? | Yes, the hospital management played a crucial role in participant recruitment by disseminating information about the study to the heads of departments and waste handlers in their respective departments (Page 4). |
| Can you explain the process of data collection using the structured questionnaire? How were the questions formulated and what were the categories used for data variables? | These are explained as: Data collection was conducted using a structured questionnaire comprising close-ended questions. The use of closed questions facilitated easy processing of answers, enhanced comparability, and facilitated the presentation of relationships between variables, allowing for comparisons between respondents. The questionnaire consisted of four parts, and data variables were categorized accordingly:   - Part one covered the demographics (age, gender, language, and highest qualifications). - Part two covered knowledge of isolation waste. - Part three focused on the understanding of risks associated with handling isolation waste. - Part four looked at training and policies on isolation waste. |
| How was the knowledge of waste handlers on handling isolation waste assessed? Can you provide more details about the rating scale used and the interpretation of the responses? | To assess the knowledge of waste handlers on handling isolation waste, a rating scale was used whereby responses were rated from 0-2, with 0 indicating very poor, 1 being good and 2 being very good. To respond to questions on the usage of hygiene, PPE, safety precautions and training, respondents had to select the correct answer and three or five options were listed, some questions required respondents to give a yes/no/ do not know response (Page 5). |
| What were the ethical considerations taken into account for this study? Can you elaborate on the voluntary participation and the informed consent process? | **Ethical clearance for this study was obtained from the Institutional Research Ethics Committee at Durban University of Technology, with the assigned IREC number 139/19. Subsequently, permission to conduct the study and contact the participants was sought and granted from the Gauteng Department of Health. Participation in the study was entirely voluntary, and participants had the right to withdraw at any point without facing any consequences. Those who agreed to take part were provided with consent forms and letters of information detailing the study's purpose, procedures, and their rights as participants. To ensure confidentiality, the researcher refrained from using participants' names in the report. Additionally, no information or data that could potentially identify respondents was included. The collected information was solely used for the research report and was not shared for any other purpose. Participants were also assured that their involvement in the study would not result in harm or injury. Moreover, the potential benefits of the study were explained, emphasizing its contribution to improving isolation waste management practices in healthcare facilities (Page 6).** |
| Can you provide more details on the methodology used to assess waste handlers' knowledge and understanding of isolation waste management? | The knowledge and understanding of isolation waste were assessed using structured questionnaire. All questions that allowed respondents to select more than one correct answer were assigned a score of 2 if the respondent provided multiple correct answers. For questions with single correct responses answered correctly, a score of 1 was awarded. Incorrect or negative responses were scored as 0. |
| Were there any limitations or challenges encountered during the study that may have affected the results? | None was encountered. |
| What are the implications of waste handlers' good understanding of the types of waste generated in the isolation unit and the specific examples of sharp waste? | These are provided in the discussion section. For reference: The findings revealed that a majority of waste handlers demonstrated a good understanding of the types of waste generated in the isolation unit, as well as the specific examples of sharp waste, such as injections, blades, glass slides, and needles. This is practically important as the understanding of waste handlers in the handling of isolation waste could contribute to a safer and healthier environment within the healthcare facility. Besides, the understanding the types of waste, especially sharp waste, ensures that waste handlers can take appropriate safety precautions to prevent injuries and potential exposure to harmful pathogens. Proper handling and disposal of sharp waste reduce the risk of needlestick injuries and transmission of bloodborne infections such as HIV, hepatitis B, and hepatitis C. This aligns with Sutrisno and Meilasari (16) that effective management of the waste generated is of vital importance to curb the spread of disease (Page 14). |
| How does the correct identification of the colour code used to represent infectious waste contribute to proper waste segregation and disposal practices? | According to (17), adequate waste categorization and disposal practices have implications for environmental protection. It is reasonable to agree that by correctly segregating and disposing of waste, the facility minimizes the potential negative impacts on the environment, such as soil and water contamination (18). This aligns with broader sustainability goals and waste reduction initiatives of South Africa (19). |
| Can you explain the significance of waste categorization and disposal practices for environmental protection and sustainability goals in South Africa? | The proper categorization and segregation of different types of waste, such as hazardous and non-hazardous waste, and the implementation of appropriate disposal methods, are highly significant for South Africa. These practices can minimize the release of harmful pollutants into the environment, which is crucial for protecting ecosystems and safeguarding human health (17). In addition, waste categorization and disposal practices are essential components of South Africa's efforts to achieve environmental protection and sustainability goals, as outlined in the legislative framework (19). |
| What specific training methods or strategies do you recommend for different age groups to improve their knowledge of isolation waste? | Training initiatives could include on-the-job training sessions designed for healthcare workers and waste handlers to familiarize them with isolation waste management protocols, safety procedures, and regulatory requirements. Additionally, the provision of continuing education programs or professional development courses focusing on isolation waste management topics, such as infection control, hazardous waste handling, and personal protective equipment (PPE) usage, is essential. These initiatives aim to reinforce waste management practices and promote adherence to safety protocols. |
| How do you suggest utilizing experienced waste handlers as mentors to enhance knowledge dissemination among newer staff members? | To achieve this, formal mentorship programs can be established, pairing experienced waste handlers with newer staff members based on job roles, areas of expertise, or specific needs identified during training. |
| Are there any specific recommendations or actions that you propose for Dr. George Mukhari Academic Hospital to further improve waste management practices and ensure a safe working environment? | Overall, the study recommends that Dr. George Mukhari Academic Hospital conduct regular training sessions and workshops for healthcare workers and waste handlers on proper waste segregation, handling, and disposal procedures. This includes providing education on infection control measures, instructing on the proper use of personal protective equipment (PPE), and ensuring compliance with regulatory requirements. Additionally, hospital management should regularly monitor and supervise waste management practices to ensure compliance with protocols and regulations. By following these recommendations, Dr. George Mukhari Academic Hospital can further improve waste management practices, ensure a safe working environment, and contribute to infection control and environmental protection. |
