# Supplementary material for: An investigation into waste handler’s knowledge of management of isolation waste: A case study of Dr George Mukhari Academic Hospital, Gauteng, South Africa
Source: PLoS One. 2024 Jun 10;19(6):e0305224. doi: 10.1371/journal.pone.0305224 (PMC11164387; doi:10.1371/journal.pone.0305224)
Supplement: Attachment — Submitted filename: Comments.docx [file pone.0305224.s003.docx]

The manuscript An investigation into waste handler’s knowledge of management of isolation waste: A case study of Dr George Mukhari Academic Hospital, Gauteng, South Africa has undergone substantial improvements, yet there remain several points that require attention:

1. Data collection was conducted using a structured questionnaire comprising close-ended questions. The questionnaire consisted of four parts, and data variables were categorized accordingly: This might be present in a flow chart diagram. Lines 122 – 125; Page 5
2. The reviewer strongly encourages the Authors to include the waste segregation system and collection scheme applied in the Country of study (South Africa) in the introduction section. This will add quality to their finding and justify the study.
3. The reviewer acknowledges the primary focus of the study was the investigation into waste handler’s knowledge of the management of isolation waste. However, do Authors consider the treatment of health care waste such as biological treatment, Incinerating treatment, Steam-based treatment technologies, etc. might also be a solution to the challenges in South Africa?
4. Lines: 166 – 167 “In terms of age groups, 50% were between 40-49, 23.5% each were below 20 and between 21-39, and the same for 50 and below” Please re-write this part (40-49, 23.5%) a bit difficult to understand. A similar situation is found in the result section.
5. Please be consistent with punctuation: line 176 Author wrote ((n=25, 73.5%), I think instead of (n=25; 73.5%). A similar situation is found in the result section.
6. The reviewer strongly recommends Authors improve Table 3. (Give a space in between rows associated with each list of Knowledge on isolation unit waste). It is very important to readers for clarity. For instance, Table 2. is a good example of how tables should be.
7. Please revise Table 2: Knowledge of waste handlers on different types of waste generated in isolation unit, if it is a mistake. “Knowledge of isolation waste” is presented twice in the first column.
8. Recommend Authors for more depth of discussion. For instance, in lines 299 - 300 (According to (17), adequate waste categorization and disposal practices have implications for environmental protection). Please compare your findings with some of those references in your manuscript with what exactly they do. This will increase the quality of your study.
